# New Diagnostic Criteria for Hypertension in Children and Adolescents: Lights and Shadows

**DOI:** 10.3390/children7110196

**Published:** 2020-10-24

**Authors:** Procolo Di Bonito, Anna Di Sessa

**Affiliations:** 1 Department of Internal Medicine, “S. Maria delle Grazie” Pozzuoli Hospital, 80078 Naples, Italy; procolodibonito53@gmail.com; 2 Department of Woman, Child and General and Specialized Surgery, University of Campania “Luigi Vanvitelli”, 80138 Naples, Italy

**Keywords:** hypertension, pediatric, clinical practice guidelines, blood pressure, cardiovascular risk

## Abstract

Pediatric hypertension (HTN) represents a challenging disease with a major cardiometabolic risk (CMR) burden from childhood to adulthood. In fact, it has been linked to cardiac and vascular damage even at pediatric age and recognized as an independent risk factor for HTN in adulthood. Therefore, HTN in children has gained remarkable scientific interest during the past decades. However, the availability of different diagnostic classifications complicates HTN definition. The Clinical Practice Guidelines released in 2017 updated the diagnostic criteria, by highlighting some important issues with clinical implications. Lowering the new cut-offs proposed by the CPG, as compared with those proposed by IV Report criteria, will increase the number of young people at risk of hypertension. However, evidence suggests that the CPG cutoff-points in further identifying subjects with an altered CMR profile. Currently, some issues are still debated such as the adoption of a fixed cut-off of BP ≥ 130/80 mmHg for children aged ≥ 13 years, or the adoption of criteria for cardiac damage derived from adults. Given the CMR burden of pediatric HTN, a better and early identification of children at higher HTN risk is strictly recommended in order to improve HTN management to reduce the cardiovascular risk in these youths.

## 1. Introduction

Over the past four decades, 22,200 scientific reports regarding “Pediatric Hypertension” (HTN) have been produced since 1950 and, more importantly of which, 21,200 in the last 20 years (https://pubmed.ncbi.nlm.nih.gov/?term=pediatric+hypertension). This revealed an increased both clinical and scientific interest about pediatric hypertension, since the release of the first guidelines of the Fourth Report of National Blood Pressure Education Program Working Group on High Blood Pressure in Children and Adolescents (IV Report) [1].

In adults, HTN is defined by blood pressure (BP) levels related to development of cardiovascular events. Similar pediatric studies are still lacking. Therefore, HTN in children and adolescents is based on the assumption that 95th percentile of systolic BP and/or diastolic BP was the most suitable cut-off (from a probabilistic point of view) to identify youth at high risk to develop HTN. However, it should be noted that the diagnosis of pediatric HTN is based on complex tables derived from a database of National Health and Nutrition Examination Survey (NHANES) containing both systolic and diastolic BP percentiles, closely linked to age, sex, and height [1]. These cut-offs proposed by IV Report, unchanged from 2014 to 2017, identified four categories of BP, for age, sex, and height:

- Normal BP (BP < 90th percentile);
- Pre-hypertension (BP ≥ 90th < 95th percentile);
- Stage 1 hypertension (BP ≥ 95th < 99th +5 mmHg);
- Stage 2 hypertension (BP ≥ 99th +5 mmHg).

In 1996, the European Society of Hypertension (ESH) updated the IV Report but no changes were made for diagnostic criteria for patients up to 16 years. Instead, for subjects aged ≥ 16 years, a novel fixed criterion (independent of age and sex) as BP ≥ 140/90 mmHg was introduced [2]. Taken together, these intricate procedures have discouraged pediatricians in a correct BP interpretation in clinical practice according to IV Report. This has been confirmed in several retrospective studies based on electronic database that showed a large underestimation of diagnosis of HTN [3,4], also worsened by the need to confirm elevated BP levels detected at first observation in two following occasions [1,2]. These procedures might discourage both pediatricians and families to further investigate youths at higher risk of hypertension, resulting in a potential underestimation of HTN diagnosis.

### New Guidelines

Following the publication of the new Clinical Practice Guidelines (CPG) released by the American Academy of Pediatrics in 2017, pediatric HTN has been aroused a renewed interest [5].

The main novelties were:

- Novel percentiles based on cut-offs obtained from the original database of the IV Report after exclusion of both overweight and obese individuals;
- Use of “elevated BP” instead of “pre-hypertension”;
- Simplified table;
- A diversified definition in subjects according to the age as reported in the table. In children aged < 13 years, it is recommended to use as cut-off the BP levels ≥ 95th percentile for age, sex, and height, while in those aged ≥ 13 years a fixed cut-off ≥130/80 mmHg independent of age, sex, and height (as specified in the Table 1).

This novel procedure reduced diagnostic cut-offs by an average of 1–3 mmHg in children and >4 mmHg in adolescents, respectively. Consequently, the CPG diagnostic criteria led to an increased prevalence of patients at risk of hypertension compared to the previous criteria. As expected, this generated an intense scientific debate, in particular with the European researchers as drafters of ESH guidelines [6,7,8].

After publication of CPG, several studies compared the prevalence of HTN estimated with IV Report criteria as compared to new CPG. As expected, HTN prevalence defined by IV Report was lower than the estimate calculated with CPG cut-offs. Particularly, a large study conducted in 50,336 Chinese children and adolescents showed a prevalence of 4.7% in normal weight (NW) youths, of 11.1% in overweight (OW), and of 25.5% in obese (OB) individuals, while the prevalence was of 5.8% in NW, of 13.8% in OW, and 34% in OB subjects according to the new diagnostic criteria [9].

Given the higher intrinsic risk of HTN of youths with OW/OB, our group also aimed to investigate the HTN prevalence in the CARITALY cohort including 6137 children and adolescents with OW/OB. We found a prevalence of 30.7% by using ESH criteria and of 34.8% with CPG [10]. A higher HTN prevalence in youths at risk of HTN estimated with the new criteria was also demonstrated in other studies [11,12,13]. However, prevalence studies presented some issues that should be underlined:

- The prevalence is largely influenced by different factors such as age and weight status (lower in NW, intermediate in OW, and higher in OB subjects);
- It is also influenced by the population setting (lower in population-based studies, higher in those conducted in outpatient youths)
- The majority of the studies comparing prevalence with the aforementioned two classifications analyzed data from a BP measurement in a single occasion.

This latter point represents a crucial issue because the HTN prevalence decreases within the increasing visit number [14]. It could be explained by the “white coat” HTN, a condition closely related to the stress that may occur at first BP measurement in children. Taking into account these limitations, some studies examined whether CPG criteria were more sensitive that IV Report in identifying a larger number of patients at higher risk of HTN or whether this higher percentage was also related to a worse cardiometabolic risk (CMR) profile.

Sharma et al. classified 15,647 American children and adolescents from NHANES database according to both previous and new diagnostic criteria [11]. Authors found an increased prevalence of high BP from 11.8% based on IV Report criteria to 14.2% by using CPG. They further stratified the main features of 900 patients with normal BP by using IV Report, but reclassified as hypertensive according to CPG. They observed a worse CMR profile in these patients compared to 900 sex- and age-matched individuals classified as non-hypertensive with both classifications [11].

In line with this issue, our group also aimed to evaluate whether in 2929 OW/OB youth of the CARITALY Study, the reclassification of hypertension by the CPG in individuals defined as non-hypertensive by the ESH criteria, would classify differently in relation to their CMR profile. Using CPG criteria 327 youth resulted normotensive by ESH/IV Report guidelines but hypertensive with CPG. These patients were more obese and showed dyslipidemia and a higher prevalence of left ventricular hypertrophy (LVH) than normotensive subjects according to both criteria [15]. These results confirmed the previous finding from Khoury et al. [11] and highlighted the need to identify subjects with higher risk of cardiac target organ damage. In fact, the CPG criteria provide also criteria for definition of LVH. This aspect deserves mention because of the close relationship between LVH and high BP. Thus, subjects with confirmed HTN are recommended to undergo an echocardiography not only to exclude a primitive cardiac disease but also to evaluate the potential HTN-related cardiac damage. However, it should be noted that criteria proposed by CPG for LVH diagnosis are related to cutoffs commonly used in adults (i.e., LV mass ≥ 51 g/h in both sexes) [5]. In our opinion, this aspect represents a limitation. In fact, it is not clear why cut-offs used in adults were recommended, instead of the age- and sex-specific ones commonly adopted in children. In fact, in our previous study we demonstrated that hypertensive youths with OW/OB showed a high degree of concentric LVH when classified with specific LVH criteria for age and sex. On the contrary, no differences between hypertensive and normotensive individuals were reported if adult criteria were applied [5]. This finding emphasizes some aspects:

- For LVH diagnosis it should be adopted the criteria based on age and sex or a fix cut-off of 38.6 g/m ^2.7^ rather than adult criterion proposed by CPG;
- The usefulness of echocardiography as “low-cost” test but with high effectiveness for screening of primitive HTN and a better identification of subjects at greater cardiovascular risk (in case of coexistence of HTN and LVH) as well.

Of note, another limitation of CGP might be represented by the different BP fixed criteria based on the age of 13. As known, age acts as a central factor in pediatrics for “normality” definition in the disease context, especially in case of BP levels at the transitional age from prepubertal to pubertal status. This step is well-known and associated to several pathophysiological changes including growth, hormones, glucose metabolism, and BP levels with consequent influence on cardiovascular system.

In view of the potential cardiac damage in this age-group, BP levels in youths aged 13–16 years need to be carefully confirmed using ambulatory blood pressure monitoring and an echocardiography.

Although the debate is far from over, it must consider that pediatric HTN has recently gained remarkable scientific attention. This is particularly relevant in category of youths with high cardiovascular risk such us OB/OW, kidney diseases, prediabetes/diabetes, or dyslipidemia. These individuals might take advantage from early HTN diagnosis, that can be made readily through a simple “low-cost” and easily reproducible method.

## 2. Conclusions

It is quite clear that CPG have focused pediatric attention the long-standing concern about HTN diagnosis and management in childhood not only in the general population (with lower HTN prevalence), but in particular in specific subgroup of youths at greater cardiovascular risk. Moreover, it should be noted that the new guidelines provided tables easier to refer excluding the confounding effect of BP levels observed in youths with OW/OB. However, it remains “sub judice” the usefulness to classify criteria according to age 13, whereas the age cutoff of 14–16 years would seem to be more effective in excluding subjects across the pubertal status. It seems also highly questionable the use of fixed adult’s cut-offs for LVH, whereas diagnostic criteria derived from pediatric findings should be most appropriate.

Finally, it should be considered the dichotomy regarding the European criteria (based on ESH guidelines released in 2016 that are similar to IV Report) and the novel CPG. In fact, this might be confusing in clinical practice and discouraging for both pediatricians and families about the importance of high BP levels on early cardiovascular risk or development of future comorbidities (such as HTN). Therefore, it would be desirable a globally shared HTN classification in childhood in order to improve the understanding of pediatric HTN.

## Figures and Tables

**Table 1:** Definition of categories of normal, elevated blood pressure (BP) or hypertension, according to Clinical Practice Guidelines.

	Systolic and/or Diastolic BP	Systolic and/or Diastolic BP
Age category	<13 years	≥13 years
Normal BP	<90th percentile	<120/80 mmHg
Elevated BP	≥90th <95th percentile or ≥120 <130/<80 mmHg	≥120 <130/<80 mmHg
Hypertension Stage 1	≥95th <95th percentile +12 or ≥130 <140/≥80 <90 mmHg	≥130 <140/≥80 <90 mmHg
Hypertension Stage 2	≥95th +12 or ≥140/90 mmHg	≥140/90 mmHg
